# Further Step in the Transition from Conventional Plasticizers to Versatile Bioplasticizers Obtained by the Valorization of Levulinic Acid and Glycerol

**DOI:** 10.1021/acssuschemeng.3c01536

**Published:** 2023-06-13

**Authors:** Luca Lenzi, Micaela Degli Esposti, Simona Braccini, Chiara Siracusa, Felice Quartinello, Georg M. Guebitz, Dario Puppi, Davide Morselli, Paola Fabbri

**Affiliations:** † Department of Civil, Chemical, Environmental, and Materials Engineering (DICAM), Università di Bologna, Via U. Terracini 28, 40131 Bologna, Italy; ‡ National Interuniversity Consortium of Materials Science and Technology (INSTM), Via G. Giusti 9, 50121 Firenze, Italy; § BIOLab Research Group, Department of Chemistry and Industrial Chemistry, Università di Pisa, Via G. Moruzzi 13, 56124 Pisa, Italy; ∥ Institute of Environmental Biotechnology University of Natural Resources and Life Sciences Vienna, Department of Agrobiotechnology, IFA-Tulln, Konrad-Lorenz-Strasse 20, 3430 Tulln an der Donau, Austria

**Keywords:** polymer additives, plasticizers, bio-based, levulinic acid, glycerol, valorization of waste

## Abstract

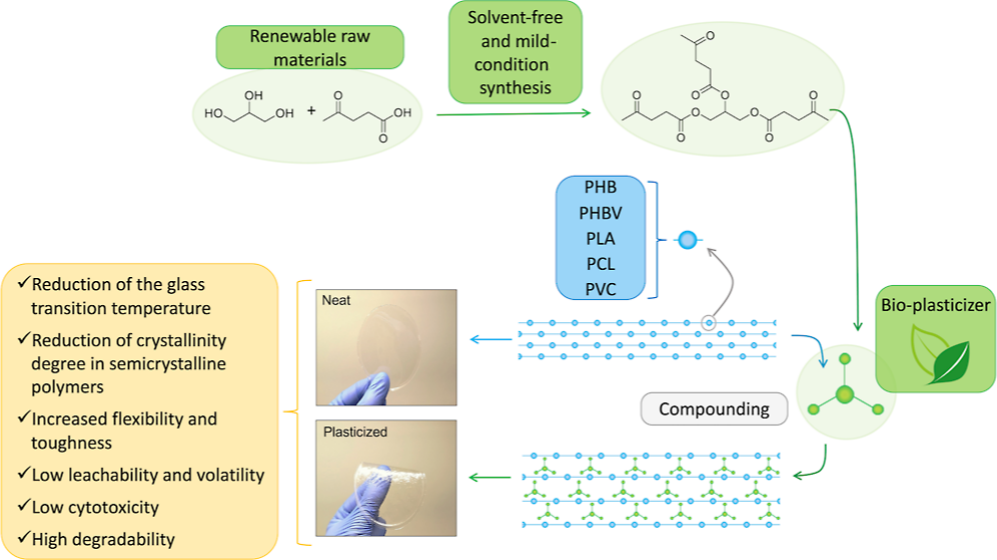

In the last two decades, the use of phthalates has been restricted worldwide due to their well-known toxicity. Nonetheless, phthalates are still widely used for their versatility, high plasticization effect, low cost, and lack of valuable alternatives. This study presents the fully bio-based and versatile glycerol trilevulinate plasticizer (GT) that was obtained by the valorization of glycerol and levulinic acid. The mild-conditions and solvent-free esterification used to synthesize GT was optimized by investigating the product by Fourier transform infrared and NMR spectroscopy. An increasing content of GT, from 10 to 40 parts by weight per hundred parts of resin (phr), was tested with poly(vinyl chloride), poly(3-hydroxybutyrate), poly(3-hydroxybutyrate-*co*-3-hydroxyvalerate), poly(lactic acid), and poly(caprolactone), which typically present complicated processability and/or mechanical properties. GT produced a significant plasticization effect on both amorphous and semicrystalline polymers, reducing their glass-transition temperature and stiffness, as observed by differential scanning calorimetry measurements and tensile tests. Remarkably, GT also decreased both the melting temperature and crystallinity degree of semicrystalline polymers. Furthermore, GT underwent enzyme-mediated hydrolysis to its initial constituents, envisioning a promising prospective for environmental safety and upcycling. Furthermore, 50% inhibitory concentration (IC_50_) tests, using mouse embryo fibroblasts, proved that GT is an unharmful alternative plasticizer, which makes it potentially applicable in the biomedical field.

## Abstract 2

Valorization of glycerol and levulinic acid by a solvent-free and mild-condition reaction to obtain a high-efficiency bioplasticizer suitable for different polymers.

## Introduction

The vast majority of conventional plastics and bioplastics are compounded with a large number of harmful chemicals.^1,2^ In particular, various conventional polymer additives do not meet the requirements in terms of renewability, biodegradability, and cytotoxicity that nowadays have become necessary.^2^ Plastic compounding has enormously changed the world of conventional plastics, offering a very effective and simple approach for tuning polymer properties according to specific applications. Also, the biopolymer sector has taken advantage of this approach for overcoming the disadvantageous mechanical, viscoelastic, and thermal properties, which limit the processability of several biopolymers^3^ and their diffusion in the everyday life. Moreover, biopolymers are often compounded with conventional additives, altering irremediably their renewable origin and/or biodegradability and/or biocompatibility. This underlines the actual need of a new generation of sustainable additives suitable for a large range of polymeric materials. Plasticizers represent worldwide more than 50% of the additives sector,^4^ offering a simple method to tailor the mechanical, viscoelastic, and thermal properties of polymers. Currently, approximately 65% of the plasticizers are derived from phthalate anhydride.^4^ This class of plasticizers, known as phthalates, has become widely used for three main reasons: low price, high plasticization effect, and versatility toward different polymers. However, it has been reported that phthalates can leach out from plastics and get dispersed in the land and sea,^5^ producing environmental pollution and severe health issues.^6−9^ In this regard, United States,^10^ Canada,^11^ and European Union,^12^ among others, have strictly regulated and in some cases banned the use of phthalates in several products (food packing, biomedical devices, and children toys) due to their demonstrated endocrine-disrupting^13^ and potential carcinogenic^9,14^ effects. Recently, alternative green plasticizers such as alkyl citrates, adipates, and epoxidized vegetable oils (EVOs) have been proposed.^15^ However, alkyl citrates and adipates cannot be compared with phthalates in terms of plasticization efficiency and versatility. EVOs have a high leaching rate, thus they cannot ensure long-lasting properties.^15^ Moreover, EVOs production is characterized by harsh conditions due to the corrosive and harmful reagents involved, making the process difficult to scale and environmentally unsustainable.^16,17^ Another well-known alternative plasticizer is 1,2-cyclohexane dicarboxylic acid diisononyl ester (Hexamoll DINCH). Recent studies have shown, also for DINCH, that long exposure can cause severe health issues.^18,19^ Furthermore, to the best of our knowledge, no study on these alternatives has shown their actual biodegradability and/or biocompatibility. For these reasons, such green alternatives have only partially replaced the use of phthalates, which instead have been having a growing demand.^20^ Typically, the design of a plasticizer is not the result of a rational molecular interaction investigation. Only recently, some more structured studies have proposed correlating plasticizers’ molecular structures and their properties.^21,22^ However, as concluded by Robin *et al.*, a lot of work is still needed to clarify aspects related not only to plasticization but also to biodegradability and biocompatibility.^23^

The current overriding need to develop new generation of additives is also underlined by specific market trends, which show that the recent intense development of biopolymers has not been correspondingly followed by the development of biodegradable and biocompatible additives from renewable resources.^24^ Analyzing the market trend of the so-called “bioplasticizers”, their developmental delay according to the paradigms based on environmental sustainability and circular economy^25^ becomes even more evident. Such observation is also supported by specific market expectations in the next five years,^26^ which clearly describe that the most significant development of new additives/plasticizers still has to take place.^27^ Combining technical considerations and market analyses, it is evident that a sustainable class of plasticizers and a new approach for their design and production are expected in the near future.^28,29^

In the last few decades, several building blocks from biowastes have been used to produce polymers, fuels, and other products.^30^ However, only a small part of the scientific community focused on the conversion of these building blocks into additives, even though they are suitable for this purpose.^31^ For instance, glycerol (GLY) is a waste from the biodiesel production process, and it is considered an excellent source for several products.^32,33^ Howell and Lazar have proposed the use of GLY to prepare hyperbranched poly(ester)s that induce only an acceptable plasticization on poly(vinyl chloride) (PVC).^34^ Marić *et al.* have recently synthesized GLY-based plasticizers for application in the food packaging field. They have studied the variations of plasticization efficiency modifying both the length and branching of alkyl side chains.^35^

Another interesting bio-based molecule is levulinic acid (LA) that has drawn special attention in the aspects of circular economy and sustainability^36^ as one of the most promising biomass-derived building block^37,38^ (from cellulose wastes) due to its large availability and relatively low price.^39^ Recently, Xuan and co-workers developed LA-based plasticizers to tailor poly(lactic acid) (PLA) properties, varying both the side chains and the central structure of the plasticizer.^40,41^ Also, our group, in the latest years, has proposed an LA-based ketal–diester synthesized by a selective protecting-group-free route as plasticizers. These molecules have been tested as plasticizers in PVC^42^ and poly(3-hydroxybutyrate) (PHB),^43^ showing very promising performances compared to commercial plasticizers. Despite the high plasticization effect, several studies have reported plasticizer syntheses or extraction procedures that are not environmentally sustainable.^44−47^ The environmental improvement that derives from valorization of biomass-derived building blocks is hindered if multi-step procedures, large amounts of solvents, and/or harsh conditions are used. Therefore, the whole process has to be fully rethought to be more sustainable and to lead to a more affordable scaleup.

While numerous research studies have been focused on testing various plasticizers with specific polymers (most of them on PVC and PLA), only few of them have comprehensively characterized the proposed new molecules as plasticizers with regard to their versatility and plasticizing efficacy across various polymers. Moreover, crucial aspects such as biocompatibility and degradability have to be taken into account for a responsible design of bio-based additives.

Herein, we have proposed the synthesis of a very versatile bioplasticizer by the esterification of LA with GLY. Specifically, this was achieved by a solvent-free reaction under mild conditions and a simple workup procedure. The obtained bioplasticizer was tested on five different polymers for demonstrating its wide plasticization effect, miscibility, and biodegradability. We have selected PVC as a model polymer (the most compounded with plasticizers) and two well-established biopolyesters, namely, PLA and poly(caprolactone) (PCL). Furthermore, PHB and a related copolymer poly(3-hydroxybutyrate-*co*-3-hydroxyvalerate) (PHBV) were studied as promising and emerging biopolymers characterized by challenging mechanical properties and molten processability that therefore require plasticizers to be improved.

## Experimental Section

### Materials

LA (98.0%), GLY (≥99%), methanol (99.8%), tetrahydrofuran (THF, HPLC grade), chloroform (CHCl_3_, HPLC grade), *n*-hexane (≥95%), water (HPLC grade), sodium sulfate (Na_2_SO_4_, anhydrous, ≥99.0%), sodium chloride (NaCl, ≥99.5%), zinc sulfate heptahydrate (ZnSO_4_·7H_2_O, ≥99.0%), potassium hexacyanoferrate(II) trihydrate (K_4_[Fe(CN)_6_]·3H_2_O, ≥98.5%), Dulbecco’s modified Eagle medium (DMEM), l-glutamine, penicillin/streptomycin solution (10,000 U·mL^–1^:10 mg·mL^–1^), and calf serum were purchased from Sigma-Aldrich. Plasmocin mycoplasma elimination reagent was purchased from InvivoGen, sodium bicarbonate (NaHCO_3_, ≥99.5%) was purchased from Carlo Erba (Milan, Italy), *p*-toluenesulfonic acid monohydrate (PTSA, 98.5%) was purchased from Alfa Aesar, ethyl acetate (EtAc, 99.96%) was purchased from Fisher Chemicals, and deuterated chloroform [(CDCl_3_, 99.8 atom % D, containing 0.03 v/v % tetramethylsilane (TMS)] was supplied from VWR Chemicals. Analytical thin layer chromatography (TLC) was performed using precoated aluminum-backed plates (Merck Kieselgel 60 F254) and visualized by a solution of potassium permanganate (KMnO_4_, 0.06 M). The above-mentioned reagents and solvents were used as received without further purification.

The murine embryo fibroblast cell line Balb/3T3 clone A31 was obtained from American Type Culture Collection (ATCC CCL-163, Manassas, USA).

PHB (custom grade, *M*_n_: 106,200, *M*_w_: 425,900), PHBV (custom grade, *M*_n_: 209,300, *M*_w_: 586,000, 20 mol % of 3HV), and PCL (*M*_n_: 46,800, *M*_w_: 77,600) were purchased from Merck group. PLA (Ingeo biopolymer PLA 4060D, *M*_n_: 103,100, *M*_w_: 232,900, copolymer ratio of D to L: 12:88 mol %) was kindly provided by NatureWorks, USA. PVC (industrial grade, *M*_n_: 64,900, *M*_w_: 150,300) was provided by Resilia Srl (Italy).

Before the addition of the synthesized plasticizer, all polymers were carefully purified by reprecipitation in order to remove all possible additives or impurities, which can alter the results. In detail, PVC and PLA were initially solubilized in THF (0.67 mg·mL^–1^), whereas PHB, PHBV, and PCL were solubilized in CHCl_3_ (0.67 mg·mL^–1^). All polymeric solutions were then vacuum-filtered on Celite and precipitated in a large excess of cold methanol, according to the procedure reported elsewhere.^48^

### Synthesis of Glycerol Trilevulinate Plasticizer

The solvent-free synthesis of glycerol trilevulinate (GT) plasticizer was carried out in mild conditions by esterifying LA with GLY. In detail, the experimental procedure was as follows: GLY (3.60 g/0.0391 mol), LA (22.70 g/0.1953 mol), and PTSA (catalyst, 0.23 g/0.0012 mol) were added to a round-bottom flask with a magnetic stirrer. The reaction mixture was heated up to 110 °C for 24 h (in an oil bath), leaving the flask open. At the end of the reaction, the obtained solution was cooled down at room temperature and quenched with a saturated NaHCO_3_ aqueous solution to neutralize the excess of LA. The solution was then extracted 2 times with EtAc by a separation funnel. The extracted organic phase was washed 2 times with saturated NaHCO_3_ (aq) and once with brine (saturated NaCl aqueous solution). After drying over anhydrous Na_2_SO_4_, the residual solvent was evaporated under reduced pressure with a rotary evaporator until a yellowish viscous product was obtained.

### Sample Preparation

**Scheme 1:**
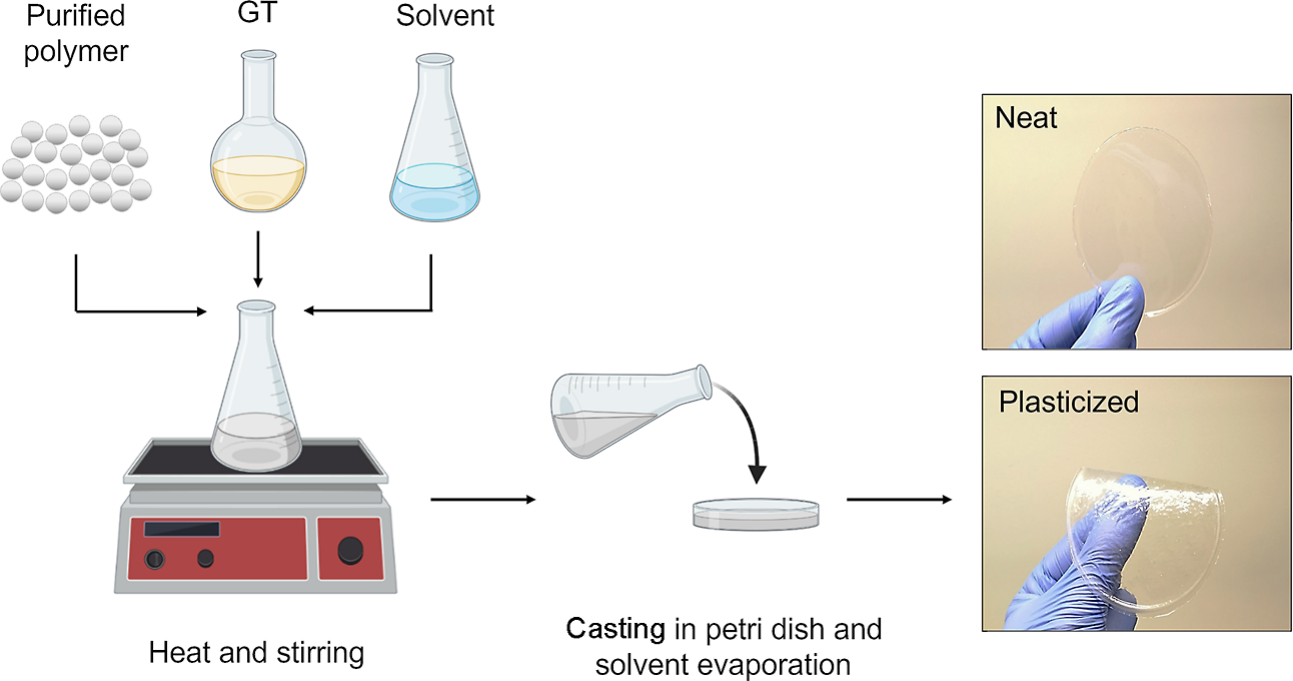
Film Preparation through a Solvent Casting Technique Representative photographs of the neat and GT compounded PVC films.

Neat and plasticized polymeric films were prepared by solvent casting as summarized in Scheme 1. Specifically, PVC and PLA were cast from THF solution (50 mg·mL^–1^), while PHB, PHBV, and PCL were cast from CHCl_3_ solution (50 mg·mL^–1^). GT plasticizer was added to the polymeric solution in 10, 20, and 40 parts by weight per hundred parts of resin (phr). The prepared solutions were cast in a Petri dish and left to dry under the hood for 24 h at room temperature. As a result, films of approximately 100 μm thickness were obtained, and no significant difference in color between neat and plasticized films was observed (Figure S1). All samples were then conditioned in a ventilated oven at 50 °C for 24 h to ensure the absence of solvents prior to testing. Neat polymeric films were used as references in all performed characterizations.

### Characterization

The reaction evolution was monitored through both TLC (using pure EtAc as the eluent) and Fourier transform infrared (FT-IR) spectroscopy. FT-IR spectra were recorded by a PerkinElmer Spectrum Two spectrometer, equipped with a diamond attenuated total reflection crystal. The selected spectral window was from 4000 to 400 cm^–1^, and 16 scans were collected for each spectrum. Spectral data were processed with Spectrum 10 software (PerkinElmer).

1D nuclear magnetic resonance (NMR) spectroscopy was used to confirm the chemical structure of the final additive. ^1^H NMR and ^13^C NMR spectra were recorded at room temperature on a Varian Mercury 400 operating at 400 MHz (nominal frequency: 399.94 MHz) for ^1^H and at 150 MHz (nominal frequency: 150.50 MHz) for ^13^C, respectively. Chloroform-*d* (CDCl_3_), containing 0.03 vol % of TMS (as the internal reference), was used as solvent. Chemical shifts (δ) were reported in ppm relative to residual solvent signals (CHCl_3_, 7.26 ppm for ^1^H-NMR; CHCl_3_, 77.16 ppm for ^13^C-NMR). The following abbreviations were used to indicate the multiplicity in NMR spectra: s, singlet; d, doublet; t, triplet; m, multiplet; br s, broad signal. All the spectra were processed with MestReNova software (Mestrelab Research S.L.).

Thermal properties such as the melting temperature (*T*_m_), glass-transition temperature (*T*_g_), and crystallinity of neat and plasticized polymeric films were measured by differential scanning calorimetry (DSC; Q10, TA Instruments), fitted with a standard DSC cell, equipped with a Discovery Refrigerated Cooling System (RCS90, TA Instruments) and under a nitrogen atmosphere (purge flow: 20 mL·min^–1^). Approximately 5 mg of each sample were cooled down to −60 or −90 °C depending on the polymeric matrix. Then, samples were first heated up to 200 °C with a ramp rate of 10 °C·min^–1^ to better highlight the melting peak, and after a quick cooling step (20 °C·min^–1^) the samples were heated a second time at 20 °C·min^–1^ in order to emphasize the glass transition. The crystallinity degree (*X*_c_) was calculated by using eq 1, where Δ*H*_m_ and Δ*H*m^0^ are the melting enthalpy of the sample obtained by DSC measurement and the melting enthalpy of the 100% crystalline polymer. Selected enthalpy of melting for 100% crystalline PHB and PCL were, respectively, 146 and 139 J·g^–1^.^49,50^
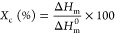
1

DSC curves were processed with TA Universal Analysis 2000 software (TA Instruments) to extrapolate the *T*_m_ and Δ*H*_m_ from the first heating scan and the *T*_g_ from the second heating scan.

Migration resistance of the plasticizer was measured by extraction tests in deionized water and *n*-hexane based on the international standard method ASTM D 1239-14.28. Approximately 100 mg each of neat and compounded polymers (containing the highest plasticizer content, 40 phr) were placed in a closed vessel containing 50 mL of extracting solvent for 24 h with gentle stirring and at room temperature. Afterward, the samples were carefully dried at 40 °C under vacuum. The mass loss due to plasticizer migration was calculated by eq 2, where *W*_1_ and *W*_2_ are the initial and final weights after the test, respectively.

The volatility of the plasticizer has been evaluated by placing the plasticized formulations in an oven in isothermal conditions. Samples of around 50 mg were stored at 70 °C for 24 h. Volatility is expressed in terms of weight loss by eq 2, where *W*_1_ and *W*_2_ are the initial and final weights after the test, respectively.
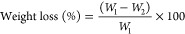
2

For both volatility and migration tests, the weight loss contributions of the neat polymers (references) were subtracted from the experimentally obtained values for the plasticized formulations.

Tensile tests were carried out to evaluate the mechanical properties of the neat and compounded materials. Tests were conducted on an INSTRON 5966 testing machine equipped with a 10 kN load cell and pneumatic grips. The speed of the crosshead was set at 4 mm·min^–1^. Samples have been prepared by cutting films in a rectangular shape of 10 mm width and 700 mm length. The length between gauges was 20 mm. Three specimens were measured for each sample.

The morphology of the samples and compatibility between the polymers and the plasticizer were evaluated by field emission scanning electron microscopy (FE-SEM), analyzing the cross-sections of the polymeric films. The cross-section of each sample was prepared by cryo-fracturing the film into liquid nitrogen. The so-obtained cross-sections were then coated with gold (thickness 10 nm) by an electrodeposition method to impart electrical conduction. Investigations were conducted using a Nova NanoSEM 450 electron microscope (FEI Company, Bruker Corporation), applying an accelerating voltage of 10 kV. The obtained images were analyzed by ImageJ open-source software.

### Enzymatic Degradation Tests

Due to the presence of ester bonds in GT, decomposition was assessed with an esterase known to hydrolyze larger molecules including polyesters, namely, a cutinase.^51^ Briefly, 3 μL (final concentration of 0.15 v/v % of bioplasticizer) was incubated in 2 mL of Milli-Q water or in 100 mM, pH 8 potassium phosphate buffer (KPO) in the presence of 5 μM of *Humicola insolens* cutinase (HiC). Controls were performed in the same conditions in the absence of the enzyme.^51^ The vials were incubated in a thermomixer (ThermoMixer 4536, Eppendorf) at 65 °C with a constant speed of 400 rpm. The reaction was monitored over 24 h, at sampling time points of 2, 4, and 6 h, with a final one at 24 h. Simultaneously, controls were run in parallel, by incubating the bioplasticizer in water without further addition of enzyme. After sampling, a cooling down step was necessary to stop enzymatic activity and proceed with storage and analysis. To evaluate the extent of enzymatic hydrolysis, high-performance liquid chromatography (HPLC) was used to quantify the released products, using a HPLC instrument (Agilent Technologies, 1260 Infinity) provided with reversed-phase column C18 and 0.01 N H_2_SO_4_ mobile phase. Before loading, the samples for analysis were prepared by Carrez precipitation to remove proteins. Potassium hexacyanoferrate(II) trihydrate and zinc sulfate heptahydrate were added sequentially before the centrifugation step (30 min, 14,000 rpm, and 4 °C) and subsequent filtration into HPLC vials. The linear compounds, GT components, LA, and GLY, were analyzed through a refractive index detector and quantified by relating to each calibration curve. These were built by plotting the refraction signal of dilution series of standards. For each monomer, the concentrations ranged from 0.1 to 20 mM, including intermediate concentrations of 0.25, 0.5, 0.75, 1, 2.5, 5, 7.5, and 10 mM.

### Biocompatibility Assessment

The mouse embryo fibroblast Balb/3T3 clone A31 cell line was employed to investigate the cytocompatibility of the plasticizer. Cells were propagated as indicated by the supplier, using DMEM supplemented with 4 mM of l-glutamine, 1% of penicillin/streptomycin solution, 10% of calf serum, and an antimycotic. The cytotoxicity study was conducted at two different time points. Balb/3T3 clone A31 cells were seeded in 96-well tissue culture polystyrene plates at a concentration of 3 × 10^3^ and 1 × 10^3^ cells per well in relation to the incubation time (24 and 72 h, respectively). After overnight incubation, the cells were treated with different concentrations (from 0 to 100 mg·mL^–1^) of the selected compound at 37 °C in a 5% atmosphere of CO_2_. A stock solution of the plasticizer in complete cell culture medium was diluted at the desired concentration. Cells incubated in the complete culture medium were used as control (CTRL). At each experimental point, cell viability was assessed by means of the WST-1 tetrazolium salt reagent assay (Roche, Basilea, Swiss). Briefly, cells were incubated for 4 h with the tetrazolium salt reagent diluted 1:10 at 37 °C and 5% CO_2_. Measurements of formazan dye absorbance, which directly correlate with the number of viable cells, were carried out with a micro-plate reader (Bio-Rad, Milan, Italy) at 450 nm, using 655 nm as the reference wavelength. The *in vitro* experiments were conducted with a single assay by testing 8 times each concentration. 50% inhibitory concentration (IC_50_) was defined as the compound concentration at which 50% of cell viability was observed in comparison to the CTRL. Cells cultured in growth medium not containing the plasticizer were considered as positive control. Cell viability *vs* concentration curves were generated by nonlinear regression (Origin 2021 software, dose–response curve), and the data were reported as mean ± standard deviation. Statistical significance of the obtained differences was determined by *t*-test analysis or one-way analysis of variance (ANOVA) with post hoc Tukey’s test. A *p* value < 0.05 was considered statistically significant.

## Results and Discussion

**Figure 1:**
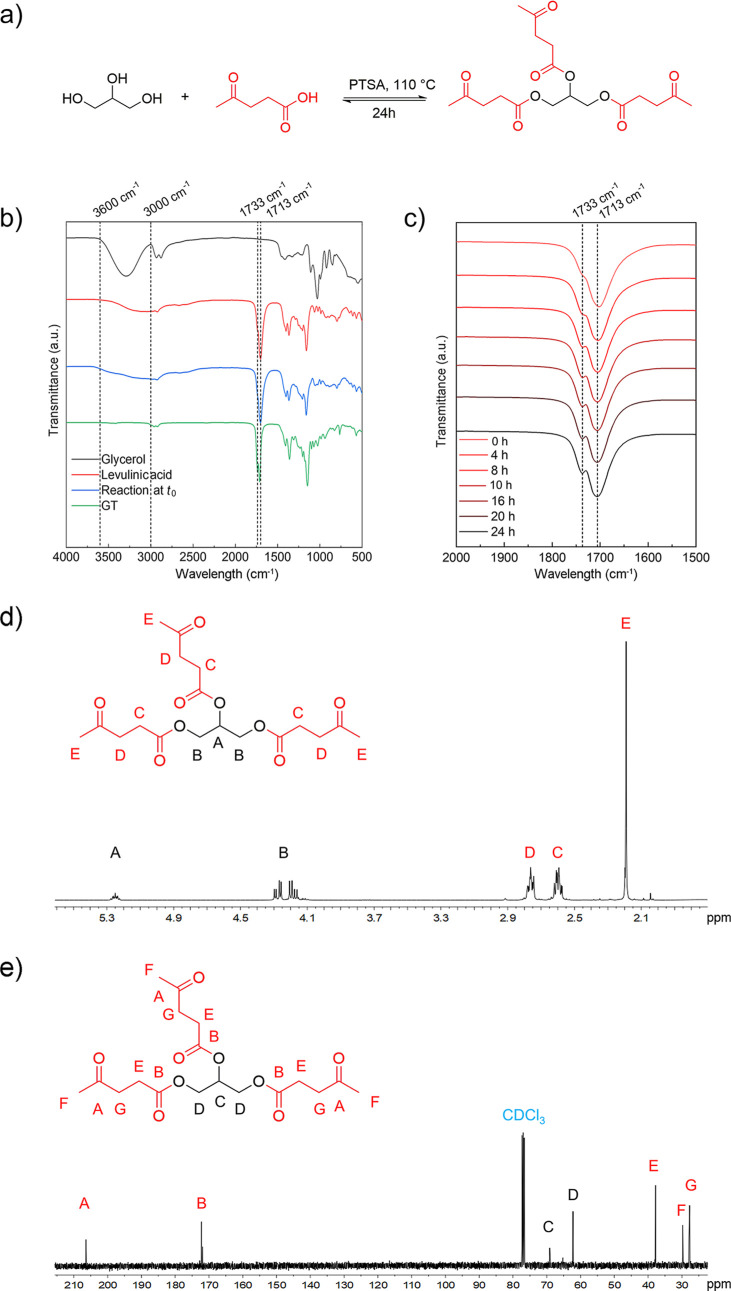
(a) Reaction scheme of the synthesis of GT plasticizer. (b) Comparison of FT-IR spectra of the reagents, the reaction mixture at *t*_0_, and the final product. Dashed lines indicate the peaks discussed in the related paragraph. (c) FT-IR spectra of the reaction mixture at different time points. Dashed lines indicate the peaks associated to the formation of the ester bond. (d) ^1^H-NMR spectrum of GT plasticizer and the corresponding proton signal assignments. (e) ^13^C-NMR spectrum of GT plasticizer and the corresponding carbon signal assignments.

GT was synthesized by solvent-free esterification of LA catalyzed by PTSA, following the reaction scheme reported in Figure 1a. In particular, a large excess of LA (LA/GLY molar ratio equal to 5) was used to increase the selectivity of the desired product, since, as previously reported by our group, with stoichiometric amounts of LA and GLY, the reaction may lead to undesired ketalization reactions.^42^ The optimum PTSA/GLY molar ratio was 0.03. The esterification was performed at 110 °C for 24 h using an open flask in order to remove water (secondary product) from the reaction environment and thus pushing the synthesis toward the desired product (GT). The above-described conditions ensured the complete consumption of GLY as shown by TLC (Figure S2) and a final GT molar yield of approx. 70%.

Both the reaction mixture and the final product were analyzed by FT-IR spectroscopy. As shown in Figure 1b, the typical broad absorption peak of GLY in the range 3600–3000 cm^–1^ due to the stretching of O–H bonds is partially overlapped with the O–H bonds’ signal of the LA. On the other hand, the ketonic group of LA is detected by the peak at 1713 cm^–1^ (Figure 1b,c) with no interference from the reaction mixture.^40,42^ The decrease of the OH-bond-related peak is significant for the formation of the ester (Figure 1b) and can be observed for monitoring the reaction progression. Nevertheless, the disappearance of this peak at the end of the reaction is not appreciable due to the large excess of LA, which remains in the mixture even after the complete consumption of GLY (indicated by TLC, Figure S2). No signal is present in the discussed range after the purification of the final product, revealing the efficiency of the extraction process and consequently the low presence of mono- or bi-substituted GLY molecules. Further progress of the reaction is indicated by the formation of ester bonds at 1733 cm^–1^ (Figure 1c). As reported by Xuan and co-workers, an absorption peak at 1733–1736 cm^–1^ in the FT-IR spectrum indicates the existence of new ester bonds.^40^ The presence of the already mentioned strong absorption peak at 1713 cm^–1^ is indicative of the presence of ketone groups, which are not involved in undesired ketalization reactions that may occur. Moreover, the absence of detectable peaks at 1100, 1090, and 960 cm^–1^ (alkoxy C–O stretching vibrations) validates the assumption that no significant ketalization reaction took place.^42^

The chemical structure of GT was investigated by ^1^H and ^13^C-NMR analyses, and the recorded spectra are shown in Figure 1d,e, respectively (detailed assignments are reported in the Supporting Information). In particular, the central structure of the molecule, which is given by the GLY, produces two multiplets centered at 5.26 and 4.23 ppm in the ^1^H-NMR spectrum (Figure 1d).^40^ The multiplets at 2.76 and 2.59 ppm correspond to the resonance of CH_2_ groups of the ethylenic chain of the newly grafted levulinic moieties, while the singlet at 2.19 ppm is related to the three protons of the ketonic groups.^40^ Low-intensity proton signals with chemical shifts δ = 5.09 ppm and δ = 4.11 ppm are attributed to the O–H groups of the partially unreacted GLY molecules (Figure S3). Integral values of the proton signals reveal a high conversion of the GLY molecules to 90% (Figure S3). As shown in Figure 1e, the signals centered at 5.09 and 4.11 ppm can be assigned to the hydroxyl groups bonded to the carbon atoms C_C_ and C_D_, respectively.

**Figure 2:**
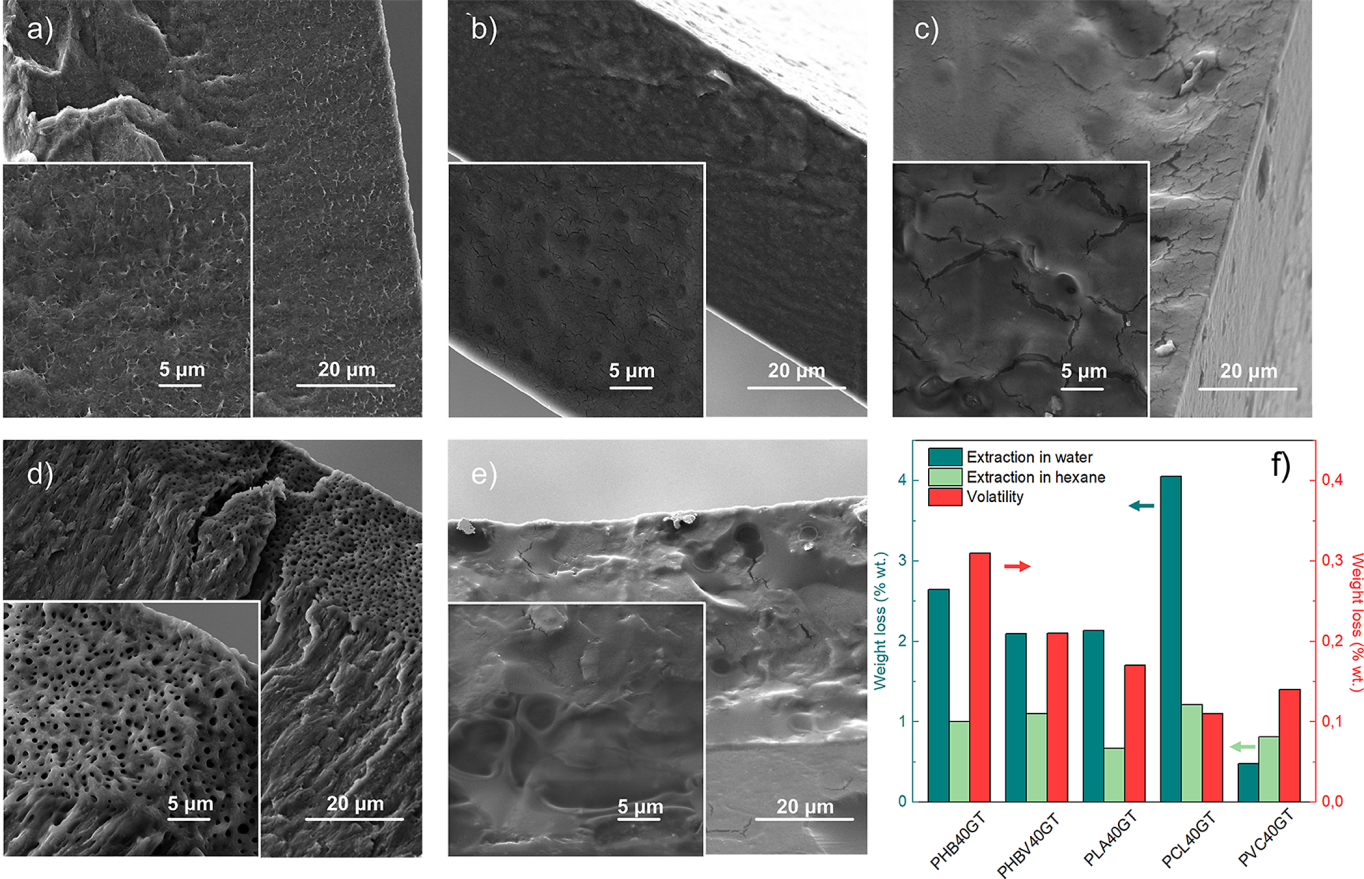
Cross-sectional FE-SEM micrographs of (a) PHB40GT, (b) PHBV40GT, (c) PLA40GT, (d) PCL40GT, and (e) PVC40GT. Higher magnifications are shown in the insets. (f) Weight loss (%) of plasticized films with 40 phr of GT calculated by eq 1 after 24 h of extraction test (room temperature) in water (dark green) and *n*-hexane (light green). Weight loss (%) obtained from volatility tests (70 °C for 24 h) is reported as red bars.

SEM analyses were used to investigate the polymer/plasticizer miscibility, which is a crucial parameter to achieve high plasticizing efficiency, low migration, and thus a long service life. It was observed that phase separation occurs due to agglomeration of additive droplets, when the solubility of a plasticizer in a given polymer is not adequate.^42^ The phenomenon occurs when the limit of solubility between the plasticizer and the polymer is exceeded, thus the insolubilized excess of the additive separates and aggregates into drop-like spots. This effect can lead to the disruption of material integrity, migration of the additive to the polymer surface, and the consequent loss of the desired thermal and mechanical properties. GT plasticizer seems to be properly solubilized within the PHB matrix even at high concentrations (20 and 40 phr), as shown in Figure 2a. PHB samples have shown no sign of phase separation with no significant morphological differences between the neat and compounded polymers. In general, at low concentrations (up to 20 phr), GT seems to have high solubility in all tested polymers (Figure S4). On the contrary, phase separation can be observed for the PHBV40GT formulation (Figure 2b), where drop-like spots of 1.5 μm in diameter are evidence of the solubility limit reached with this additive content. The same phenomenon is also detected in the PCL20GT (Figure S4), PLA40GT (Figure 2c), and PVC40GT (Figure 2e) formulations, with spots ranging from 1.5 to 10 μm in diameter. The PCL40GT formulation exhibited an anomalous behavior compared to the others (Figure 2d). The tested films presented pore-like structures of around 20 μm in size spread into the bulk of the material, which represent a clear symptom of poor miscibility.^52^

The stability of the compounded samples can be determined in terms of resistance to extraction caused from either polar or non-polar solvents. With the purpose of studying this phenomenon, neat samples and formulations containing 40 phr of plasticizer have been gently stirred for 24 h at room temperature in deionized water and *n*-hexane. Neat polymers showed a negligible leaching of always under 0.75 wt %, that can be associated with the loss of low *M*_w_ fractions. In general, a low leachability of GT has been observed in both solvents and for all polymers, as reported in Figure 2f. However, due to its polarity, GT plasticizer has shown a higher extraction in water compared to *n*-hexane, exhibiting a weight loss that ranged from 0.50 to 4 wt %. In this medium, GT shows the same affinity for short aliphatic-chain polyesters such as PHB, PLA, and PHBV (average loss, 2.3 wt %) and slightly higher loss for the PCL sample (4 wt %), which has a longer linear backbone of five carbon atoms. The plasticizer molecular structure bears two carboxyl groups in each side chain (structure in Figure 1a), which can interact with other polar groups in the polymeric chains. It is reasonable to assume that the higher the density of polar groups in the structure of the polymer, the higher will be the number of interactions with carboxyl groups of GT.^43,53,54^ The aliphatic chains of PCL do not have strong polar interactions with the plasticizer, which can explain the reason why GT has shown the tendency to be extracted by a polar solvent^40,55^ when it is compounded with this matrix. PVC40GT weight loss values, around 0.5 wt %, further confirm the hypothesis that the polarity of carbonyl groups of GT is the key factor that influences the additive leachability. This phenomenon is thus inhibited in PVC since strong dipole–dipole interactions can be established between the carbonyl groups of GT (both ester and ketonic groups) and the chlorine atoms of the polymeric chains.^42^ These interactions, stronger than the carbonyl–carbonyl ones, limit the GT mobility, which is instead entrapped among the macromolecular chains. Moreover, the polarity of GT is confirmed by the neglectable weight loss of the samples treated with *n*-hexane, never higher than 0.5 wt % for every formulation. As reported by Halloran and colleagues,^56^ a higher carbon chain length in the structure of the additive disadvantages the tendency of a plasticizer to migrate through the polymeric matrix, but it also enhances the miscibility with non-polar solvents. A possible explanation is that with increasing number of carbon atoms of the repeating unit, the density of the polar carboxyl groups decreases and so does the polarity of the compound. By this assumption, it is possible to suppose that the low solubility of GT in an aliphatic medium is hindered by the chemical incompatibility between the carbon linear chains of *n*-hexane and the carbonyl dense structure of the additive. Therefore, during the design of a plasticizer, a compromise between the chain length and the carbonyl density must be considered. The overall GT loss is acceptable with all tested polymers and in a wide range of environments. Even though the length of the chains is important in inhibiting the leaching of a plasticizer, the branched structure of the additive also plays an important role in terms of limiting the migration.^56^

Another important parameter to evaluate, in terms of environmental hazard,^57^ is the so-called volatility. The permanence of the additive inside the polymeric matrix is a function of the vapor pressure of the molecule, which is affected by the intrinsic characteristics such as the molecular mass, structure, polarity, and the ability to establish hydrogen bonding.^54,58^ For all the plasticized formulations with the highest GT content (40 phr), the weight loss was constantly lower than 0.3 wt % as reported in Figure 2f. The permanence of GT can be attributed to its branched structure^59^ and the several interactions that the molecule is able to establish with the matrix through its functional groups (aliphatic, ketonic, and ester groups).

Polymer thermal properties are typically affected by the addition of a plasticizer. In particular, *T*_g_ reduction is one of the main parameters that is used to evaluate the plasticization effect. However, our group has recently reported that the addition of a suitable plasticizer can also decrease the *T*_m_ in a semicrystalline polymer.^43^ Films of neat PHB, PHBV, PLA, PCL, and PVC and their compounds with the synthesized plasticizer were tested by DSC to investigate how the additive affects the polymer thermal properties. The obtained results were extrapolated from DSC thermograms (in Figure S5) and are summarized in Table S1 in the Supporting Information.

**Figure 3:**
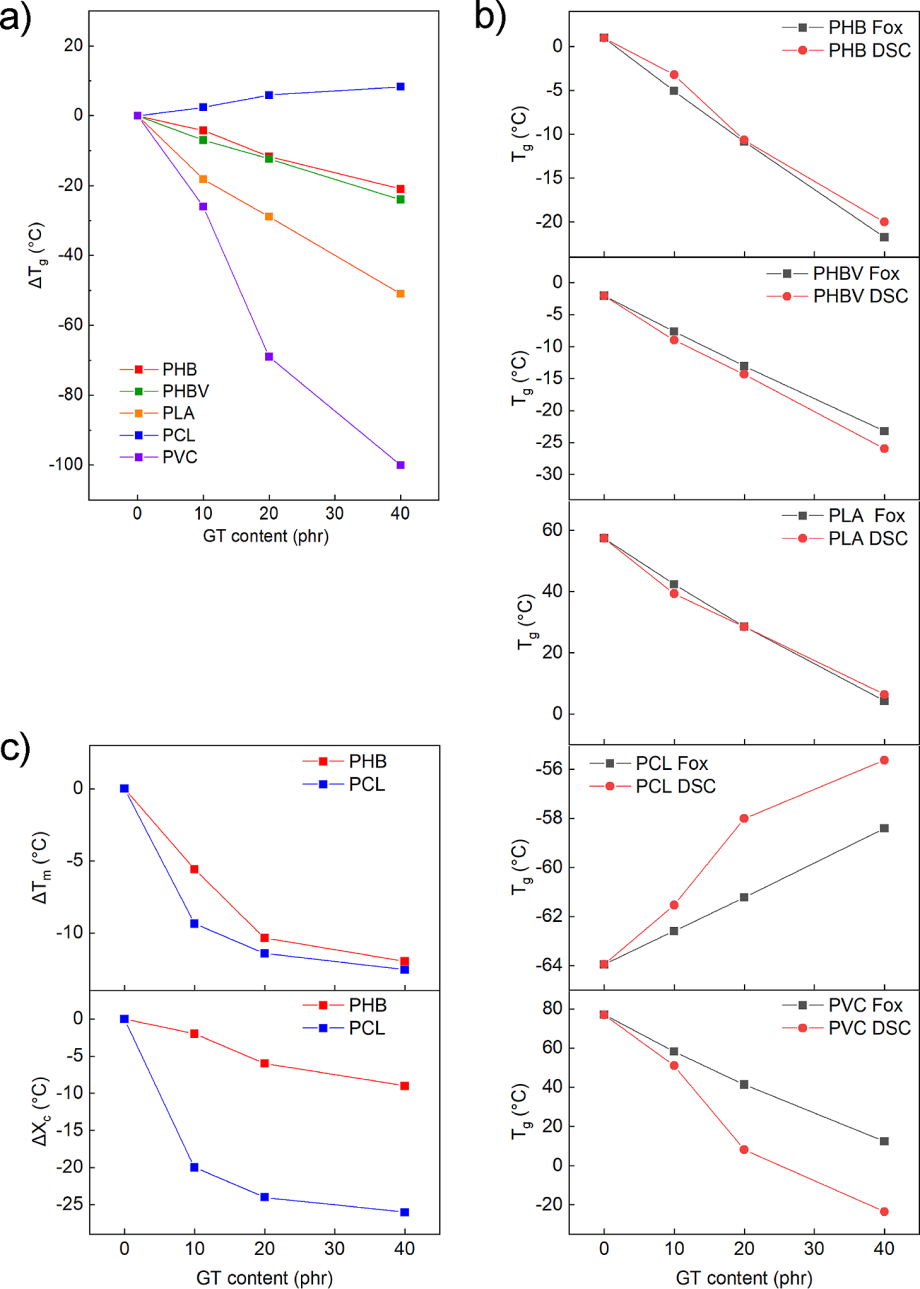
(a) Δ*T*_g_ as a function of plasticizer content for all prepared formulations. (b) *T*_g_ calculated from eq 3 (*T*_g,FOX_) and from DSC investigation (*T*_g,DSC_) plotted as a function of plasticizer content. (c) Δ*T*_m_ and Δ*X*_c_ of PHB and PCL formulations as a function of plasticizer content.

In Figure 3a, Δ*T*_g,DSC_ values calculated with respect to the neat polymer *T*_g_ are plotted as a function of the plasticizer content. As a consequence of the increase of GT content, it is possible to appreciate that Δ*T*_g,DSC_ values decrease in each formulation, indicating an efficient plasticization effect. The *T*_g,DSC_ of PHB was decreased from 1 up to −20 °C, increasing the additive content from 0 to 40 phr. A similar trend was also observed for PHBV, PLA, and PVC, in which the *T*_g_ is decreased from −2 to −26 °C, from 57 to 6 °C, and from 77 to −23 °C, respectively. The plasticization effectiveness is related to the capacity of the plasticizer to intercalate among polymeric chains, weakening their interactions and reducing the intermolecular frictions that are considered responsible for the material stiffness, as described by the lubricity theory.^60,61^ For PVC-plasticized samples, the transition from the glassy to the rubbery state is broad over a wide temperature range as shown by DSC thermograms in Figure S5e. This phenomenon complicates the detection of a discrete *T*_g_ since the midpoint of the transition is not easily detectable as is in neat PVC (Figure S5e). Considering that *T*_g_ is described as the temperature range over which segments of the polymeric backbone start moving,^62^ the observed wide transition range means that the plasticizer is able to lower the energy required to disjoint the interactions between the PVC chains at low temperatures, even if the concentration of GT is relatively low, in good accordance with what has been previously reported.^42^ The *T*_g_ decrease was not detected for the plasticized PCL samples. On the contrary, a slight increase of this parameter was observed. In order to investigate this unexpected tendency, DSC analyses were performed on bare GT, the thermogram of which is reported in Figure S6. From this test, it is possible to appreciate that the plasticizer is stable up to 200 °C without any appreciable signal due to evaporation transition, in accordance with the low volatility described previously. Interestingly, we have noticed that the plasticizer presents its own *T*_g_ of approx. −49 °C evaluated with a scanning rate of 20 °C·min^–1^. This value is higher than the *T*_g,DSC_ of PCL (about −60 °C) and thus may explain the almost linear increase of *T*_g_ of the plasticized formulations.

To further explore this hypothesis, theoretical *T*_g_ values were calculated by the Fox equation,^63^ expressed as eq 3.
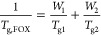
3where *T*_g,FOX_ is the theoretical *T*_g_ of the formulation. *W*_1_, *T*_g1_ and *W*_2_, *T*_g2_ are the weight fractions and *T*_g_ values of the plasticizer and the polymer, respectively. This experimentally derived relationship is useful for estimating the *T*_g_ of mixtures, knowing the properties of the pure components. The results of experimental *T*_g,DSC_ and theoretical *T*_g,FOX_ for each formulation are plotted in Figure 3b. It is possible to notice that all Fox-predicted *T*_g_ values for PHB, PHBV, and PLA formulations fit quite well to the experimental *T*_g,DSC_. *T*_g,DSC_ values of PCL and PVC show a similar trend as the calculated *T*_g,FOX_; however, divergency between the two curves is observed, especially for high additive contents (20 and 40 phr). The discrepancy between measured and theoretical *T*_g_ values is a very useful tool to understand the miscibility between the two compounds and if the plasticizer undergoes phase separation.^64^ In this regard, the comparison between Fox predictions and experimental *T*_g_ values supports the hypothesis that GT chemical affinity is stronger for short polyester chains.

Typically, a plasticizer induces an increase of the *X*_c_ due to the reduction of *T*_g_, as a consequence of the increase of free volume and the enhancement of the molecular mobility, which allows the polymeric chains to reorganize in ordered structures and thus crystallize.^65,66^ Interestingly, we have found that GT is also able to reduce the *T*_m_ and the *X*_c_ when added to semicrystalline polymers such as PHB and PCL (see thermograms in Figure S7). In Figure 3c are plotted the values of Δ*T*_m_ and Δ*X*_c_, calculated from the values listed in Table S1. For the PHB formulations, the *T*_m_ decreases from 175 to 169, 164, and 163 °C for the formulations at 10, 20, and 40 phr, respectively. Even though the *T*_m_ just decreased by a few degrees, this represents a big advantage considering the limited processability window of this material in the molten state. PHB has a high *T*_m_ (approx. 175 °C), which is very close to the degradation onset temperature of 200 °C, thus limiting its thermal processing.^67^ Furthermore, the plasticization with GT produces a decrease in crystallinity from 54% of the neat PHB down to 45% for PHB40GT (40 phr), which also leads to an increase of the film transparency (Figure S1). The *T*_m_ of PCL is also affected by the plasticizer, which results in a Δ*T*_m_ of −9 °C for the 40 phr formulation and *X*_c_ that decreases from 60% of the neat PCL down to 39, 36, and 33% for PCL10GT, PCL20GT, and PCL40GT, respectively. As for PHB, the reduction of *T*_m_ and *X*_c_ increases the thermal processability window of PCL as well as expand the possible applications where low crystallinity is required. The unexpected behavior of GT, compared to other plasticizers, can be related to its chemical structure, similarly to what has been previously reported by Sinisi *et al.*(43) for aromatic plasticizers. The abundance of carbonyl groups in the structure of the plasticizer can introduce more cohesive links between the polymeric chains that may prevent the alignment in ordered crystalline structures. Nevertheless, the branched structure of GT increases the free volume between the macromolecules and consequently reduce the *T*_g_, as observed by the experimental data.

**Figure 4:**
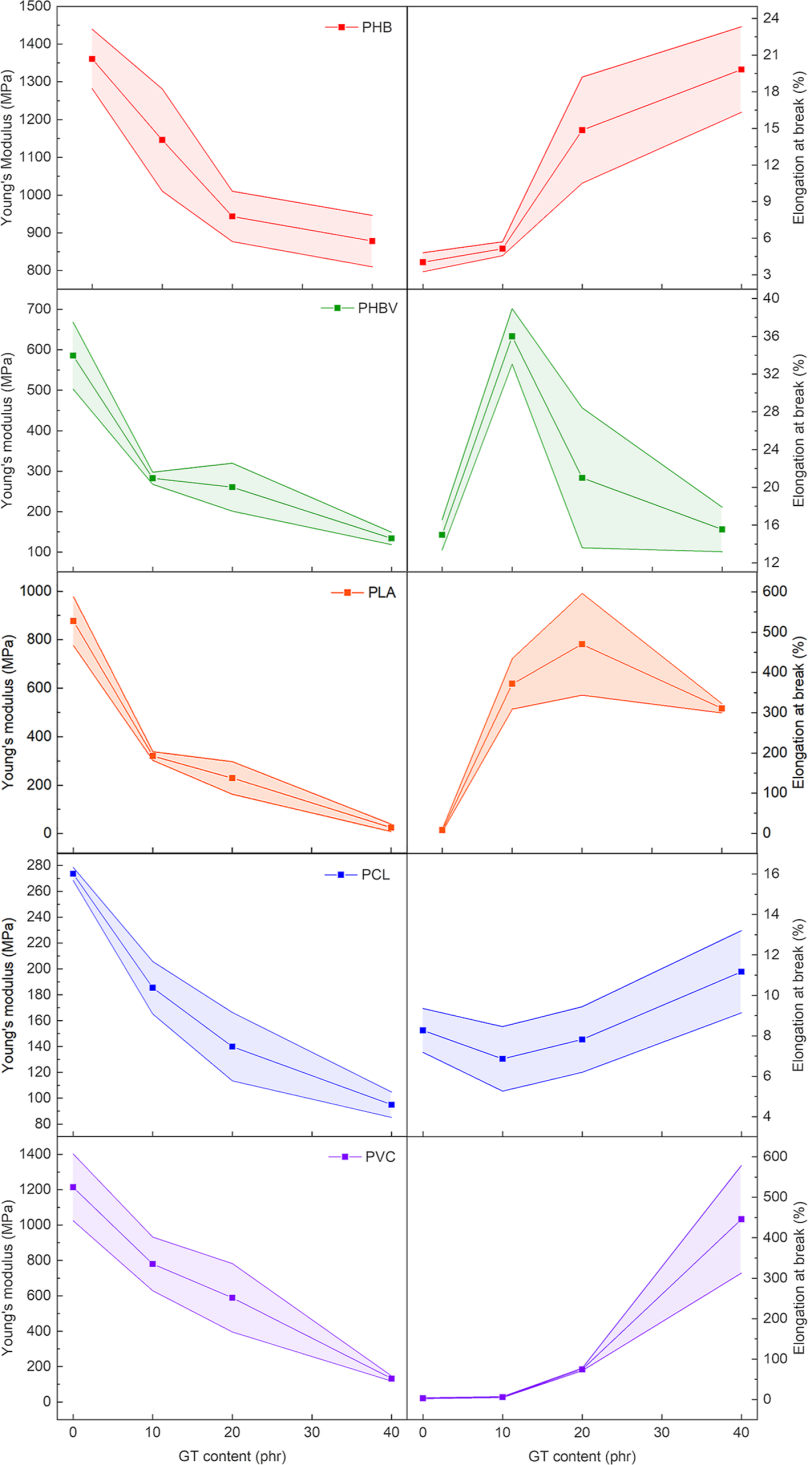
Young’s modulus (left column) and ε_break_ (right column) for each prepared polymeric compound as a function of GT plasticizer content. The shaded area of the curves represents the standard deviation of the measurements. Results of the mechanical tests are reported in Table S1 of the Supporting Information.

The addition of the synthesized plasticizer not only affects the thermal properties of the studied polymers but also their mechanical properties. As known, plasticizers can swell the amorphous phase of the polymer leading to an overall enhancement of the chain mobility and the consequent reduction of material stiffness.^54^ This increased flexibility can be noticed already at room temperature, especially when the *T*_g_ of the polymer is decreased to several degrees at room temperature. GT plasticizing effects on the mechanical properties of the selected polymers have been investigated by tensile tests (representative stress–strain curves are reported in Figure S8). The stiffness of the formulations was evaluated in terms of Young’s modulus (*E*), while the elasticity was evaluated in terms of elongation at break (ε_break_). Typically, with the increase of the plasticizer content, the polymer showed a decrease of the Young’s modulus and a consequent increment of the ε_break_. As reported in Figure 4 and Table S1, all formulations showed the expected drop of Young’s modulus, while the ε_break_ showed variable trends. In more detail, PHB showed a decrease of *E* from 1360 MPa of the neat polymer to 814 MPa for the compound with 40 phr of plasticizer, probably due to the reduction of the crystalline portion previously discussed. Meanwhile, ε_break_ increased with increasing GT content, passing from 4% for the neat sample up to almost 20% of the 40 phr formulation. PHBV samples showed a reduction in stiffness and a significant improvement of the ε_break_ (approx. 26%) up to a GT concentration of 10 phr. A further increase of the plasticizer content resulted in a decrease of the ε_break_ probably due to the occurrence of phase separation, in accordance with the previously discussed SEM images (Figures 2 and S4). PLA showed a similar trend, with the Young’s modulus that decreased from 877 MPa of the neat formulation to 134 MPa when 40 phr of GT was added. It can be also observed that the ε_break_ drastically increased from 8% at 0 phr to 372 and 470% with 10 and 20 phr of GT, respectively, reaching a level of plasticization comparable with others promising bio-based plasticizers.^35^ Contrariwise, a decrease of the ε_break_ (311%) is observed when the GT content is further increased to 40 phr, which may be due to the PLA/GT phase separation previously detected with SEM investigations (Figure 2c). PCL formulations showed a negligible variation of ε_break_, whereas *E* was reduced from 274 to 95 MPa. The phase separation detected by SEM investigation for PCL20GT matches the loss of stiffness observed by tensile tests. The more fragile porous structure present in PCL40GT, compared with the bulk and homogeneous PCL, explains the low *E* value recorded for this formulation.^68^

Both Young’s modulus and ε_break_ of PVC are significantly affected only when the plasticizer content is above 10 phr. In particular, the ε_break_ is increased from 3 (neat PVC) to 6% for PVC10GT. Instead, for the 20 and 40 phr formulations, a remarkable increase of the ε_break_ has been found, values of which reached 74 and 445%, respectively. Despite the slight phase separation and the significant drop of *E* (around 132 MPa) of PVC40GT, an outstanding improvement of neat PVC elasticity has been achieved, outperforming the mechanical properties obtained with commercial fossil-based plasticizers.^69^ This is probably due to the strong dipole–dipole interaction between the chlorine atoms of the polymeric chains and the carbonyl groups of GT, which also preserve the mechanical stability of the material.^42^ On the other hand, an important drop of the ε_break_ of PHBV is observed when the plasticizer content is higher than 10 phr (Figure 4). This result can be explained by the inter-chain dipolar interactions between macromolecular carbonyl groups that are not strong enough to avoid the phase separation when the GT content increases over 10 phr.

**Figure 5:**
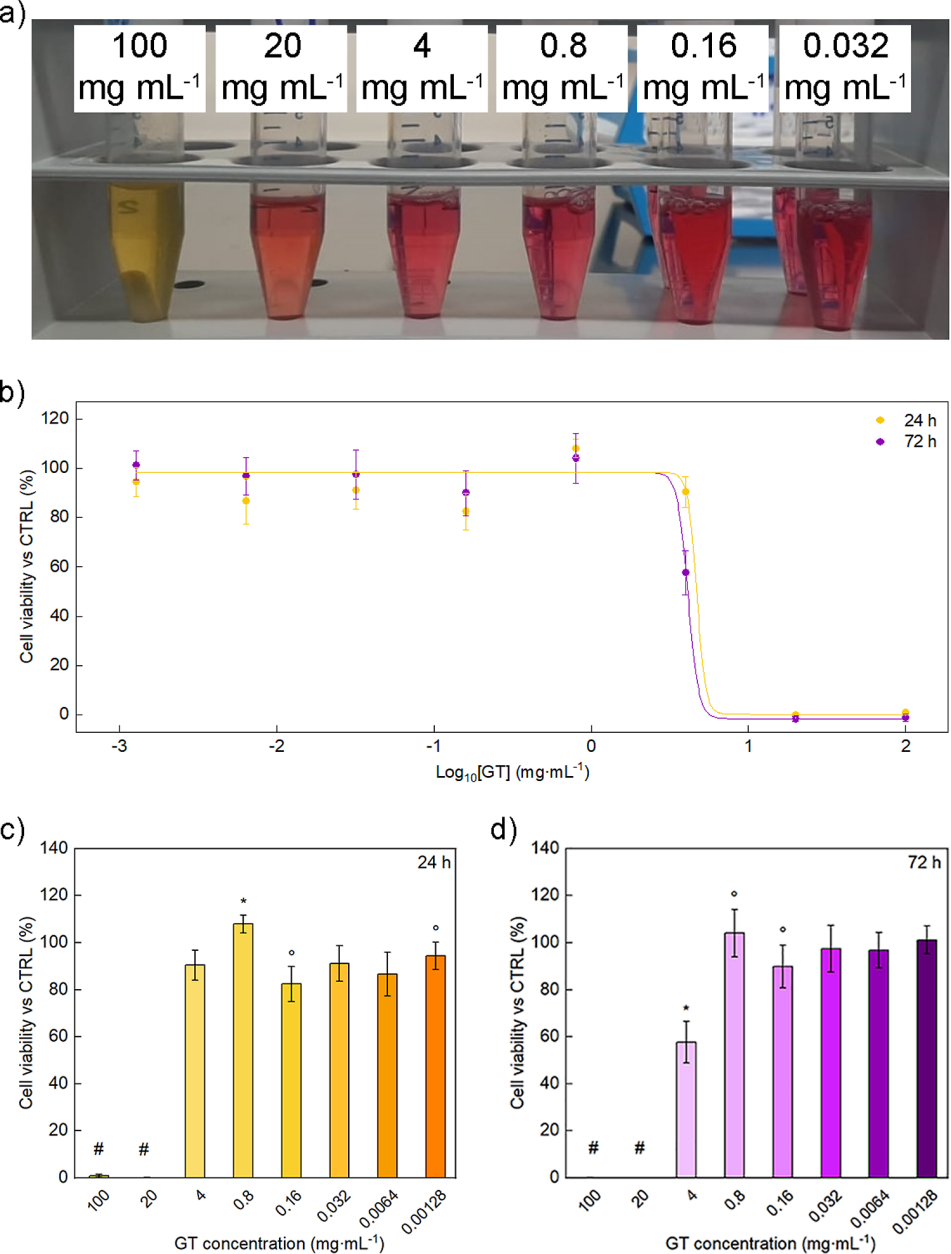
Cytotoxicity investigation: (a) representative pictures of the mixtures obtained by plasticizer dilution in cell culture medium; (b) sigmoidal curves of cell viability as a function of concentration of GT plasticizer at 24 and 72 h; cell viability expressed as percentage *vs* control (CTRL, set to 100%) at various concentrations of GT plasticizer at (c) 24 and (d) 72 h. ^#^ Values statistically significant when compared with values in the range 4–0.00128 mg·mL^–1^; * Value statistically significant when compared with the others; ° Values statistically significant when compared with each other (ANOVA).

The *in vitro* cytocompatibility of the developed plasticizer was evaluated against the Balb/3T3 clone A31 cell line. The tested plasticizer concentrations (from 100 to 0.00128 mg·mL^–1^) were obtained by serial dilutions in complete culture medium. The solutions had a pH comparable to that of the free culture medium (pH 7.4), except in the case of the most concentrated solutions showing a yellow (100 mg·mL^–1^) or an orange (20 mg·mL^–1^) color due to medium acidification (pH < 7) (Figure 5a).

The percentage cell viability curves and IC_50_ values obtained at the two investigated time points are shown in Figure 5b–d. The IC_50_ value significantly decreased with increasing incubation time (6.3 ± 2.8 and 4.1 ± 0.2 mg·mL^–1^ at 24 and 72 h, respectively) (*t*-test). However, GT resulted to be cytocompatible (cell viability ≈ 100%) at both incubation times for the concentration in the range of 0.8 to 0.00128 mg·mL^–1^, while a concentration of 4 mg·mL^–1^ resulted in a significantly lower viability at 72 h, but not at 24 h, in comparison to the positive control. Plasticizer concentrations of 20 and 100 mg·mL^–1^ resulted in cell viability values of around 0% at both experimental time points. This result could be related to the acidification of the media observed at these concentrations. However, it should be considered that the tested concentrations cover a broad range including values much higher than those commonly tested for this type of samples (*e.g.*, 0.0004–0.4 mg·mL^–1^).^70,71^ Furthermore, the IC_50_ values found in this study turn out to be considerably higher than those obtained for commercial and novel plasticizers investigated in previous studies, even if employing other types of cell lines.^72−74^ All these pieces of evidence highlight the high biocompatibility of the analyzed plasticizer. In any case, the obtained IC_50_ values should be assessed in relation to the expected maximum concentrations of the investigated plasticizer in physiological medium and in relation to the targeted application.

**Figure 6:**
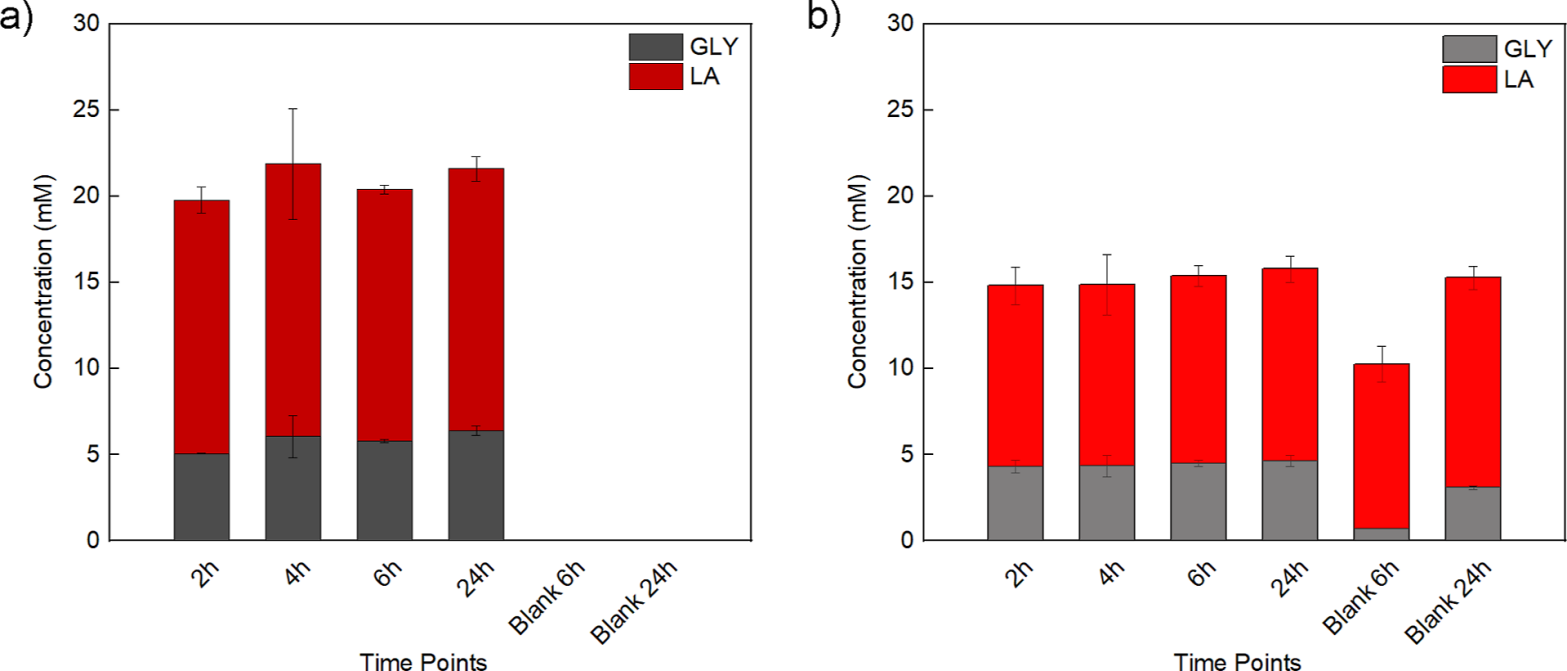
Concentration of LA (red) and GLY (gray) as a function of time, released from the enzymatic hydrolysis of GT in (a) Milli-Q water and (b) with the addition of 100 mM pH 8 KPO buffer.

In nature, biodegradation of polymers and large molecules starts with their enzymatic decomposition outside microbial cells. Thus, enzymatic hydrolysis correlates to degradation assays as it was previously shown for structurally different polyesters.^75^ Since GT bears three ester bonds, its enzymatic hydrolysis was investigated. Specifically, a cutinase, active on the polyester cutin in plants and known to also hydrolyze ester bonds in synthetic polyesters,^51^ was specifically selected to mimic potential co-biodegradation of the herein proposed plasticizer. HPLC analysis of the incubated samples with water only (Figure 6a) showed that when the enzyme is added, the ester bonds of GT are enzymatically hydrolyzed resulting in LA and GLY formation. Moreover, the enzymatic hydrolysis reached a plateau after 4 h of incubation, while no hydrolysis was observed for the blank reactions (no enzyme, Figure 6a). This effect can be also visually observed during incubation, when the control samples, incubated with water only, remained in the biphasic state with clearly distinguishable water/bioplasticizer phase separation (as shown by Figure S9a). On the other hand, the enzymatic hydrolysis led to significantly more homogeneous solutions (as shown by Figure S9b), suggesting that GT undergoes enzymatic hydrolysis.

Comparable efficiency of degradation occurred when GT was hydrolyzed in 100 mM pH 8 KPO buffer (Figure 6b). It is possible to speculate that the degradation of GT occurred in a shorter time compared to the water environment since, from the HPLC measurements, it was observed that the concentrations of GLY and LA have not been changed after the 2 h time point. It should be considered that the HPLC-based methods only quantify soluble molecules in the hydrolysate. Therefore, a slight difference in concentration may be observed between the two systems (Figure 6a,b) due to the varying solubility of LA and GLY in water or buffer. On the other hand, significant amounts of LA and GLY were also traced in the blank reactions (where no enzyme was added), showing that the ionic strength of the buffer might partially be effective for hydrolysis of the plasticizer in the above-mentioned conditions (65 °C and 400 rpm).

## Conclusions

The herein-presented work shows a valorization approach to convert GLY and LA in a versatile bioplasticizer for different polymers, representing a potential alternative to the existing “green plasticizers”. In particular, GT was obtained by solvent-free and mild-condition esterification of LA with GLY. The plasticizing efficiency of GT was tested on both fossil- and bio-based polymers such as PVC, PCL, PHB, PHBV, and PLA. We observed that an increasing content of GT not only reduces the *T*_g_ of all tested polymers but also decreases the *T*_m_ and *X*_c_ of semicrystalline polymers such as PCL and PHB. The tensile tests have shown that 10 phr of GT can already induce a significant decrease of Young’s modulus of all tested polymers, leading to a remarkable improvement of the flexibility at room temperature. Furthermore, it was observed that 40 phr of GT is the threshold of solubility for PHBV, PCL, and PVC, leading to polymer/plasticizer phase separation, especially for PHBV with the consequent drop of the ε_break_. However, volatility and migration tests (in water and *n*-hexane to simulate two environments) showed a limited weight loss of GT always lower than 0.3 and 4%, respectively. It is noteworthy that the plasticization effect obtained on PLA with only 20 phr of GT allowed us to reach an ε_break_ of 470%. With the same additive content, the *T*_m_ of PHB reduced by about 10 °C and its ε_break_ moved to approx. 15%. These results represent important achievements for a wide applicability of both PLA and PHB when a malleable material is required. Moreover, the effect on the *T*_m_ of PHB makes it possible to overcome the well-known drawback of this polymer, which has the thermal-degradation onset and *T*_m_’s very close (approx. 20 °C), thus limiting its melt-processability.

In addition, the developed plasticizer does not affect the viability of the Balb/3T3 clone A3 cell line in a wide concentration range, resulting in IC_50_ values higher than those reported in the literature for commercial and other investigated plasticizers. The GT bioplasticizer is also demonstrated to be susceptible to enzymatic (cutinase) degradation, demonstrating a potentially available upcycling. All presented results make it possible to define the herein-presented molecule as a bioplasticizer with a high plasticization efficiency toward different macromolecules.

The presented approach opens up a sustainable and responsible strategy for designing and synthesizing not only plasticizers but also other types of bioadditives, which do not alter the biodegradability, biocompatibility, and/or bio-based origin of the hosting polymer.

## Supporting Information Available

Pictures of polymeric films with different contents of plasticizer; pictures of TLC plates used to monitor the progression of the reaction; ^1^H- and ^13^C-NMR assignments of the final product; ^1^H-NMR spectrum of GT plasticizer; FE-SEM pictures of neat and plasticized films; DSC thermograms of the formulations with the corresponding *T*_g_,_DSC_; thermal properties of the formulations derived from DSC analysis; DSC thermograms of GT plasticizer; DSC thermograms of the formulations with the corresponding *T*_m_; tensile test curves of the formulations; and pictures of incubation vials with plasticizer without and with the enzyme (PDF)
