# Supplementary material for: Further Step in the Transition from Conventional Plasticizers to Versatile Bioplasticizers Obtained by the Valorization of Levulinic Acid and Glycerol
Source: ACS Sustain Chem Eng. 2023 Jun 13;11(25):9455–69. doi: 10.1021/acssuschemeng.3c01536 (PMC10302884; doi:10.1021/acssuschemeng.3c01536)
Supplement: Supplementary file 1 — sc3c01536_si_001.pdf [file sc3c01536_si_001.pdf]

SUPPORTING INFORMATION

Further Step in the Transition from Conventional Plasticizers to Versatile Bioplasticizers Obtained by the Valorization of Levulinic Acid and Glycerol

Luca Lenzi [‡,§], Micaela Degli Esposti [‡,§,*], Simona Braccini [§,†], Chiara Siracusa [#], Felice Quartinello [#], Georg M. Guebitz [#], Dario Puppi [§,†], Davide Morselli [‡,§,*], Paola Fabbri [‡,§]

[‡] *Department of Civil, Chemical, Environmental, and Materials Engineering (DICAM), Università di Bologna, Via U. Terracini 28, 40131 Bologna, Italy*

[§] *National Interuniversity Consortium of Materials Science and Technology (INSTM), Via G. Giusti 9, 50121 Firenze, Italy*

[†] *BIOLab Research Group, Department of Chemistry and Industrial Chemistry, Università di Pisa, Via G. Moruzzi 13, 56124 Pisa, Italy*

[#] *University of Natural Resources and Life Sciences Vienna, Department of Agrobiotechnology, IFA-Tulln, Institute of Environmental Biotechnology, Konrad-Lorenz-Strasse 20, 3430 Tulln an der Donau, Austria*

* Corresponding Author

Davide Morselli
Phone: +39 051 2090363
email: davide.morselli6@unibo.it

Micaela Degli Esposti
+39 051 2090363
micaela.degliesposti@unibo.it

Pages: S1 – S8
Figures: S1, S2, S3, S4, S5, S6, S7, S8 and S9
Tables: S1

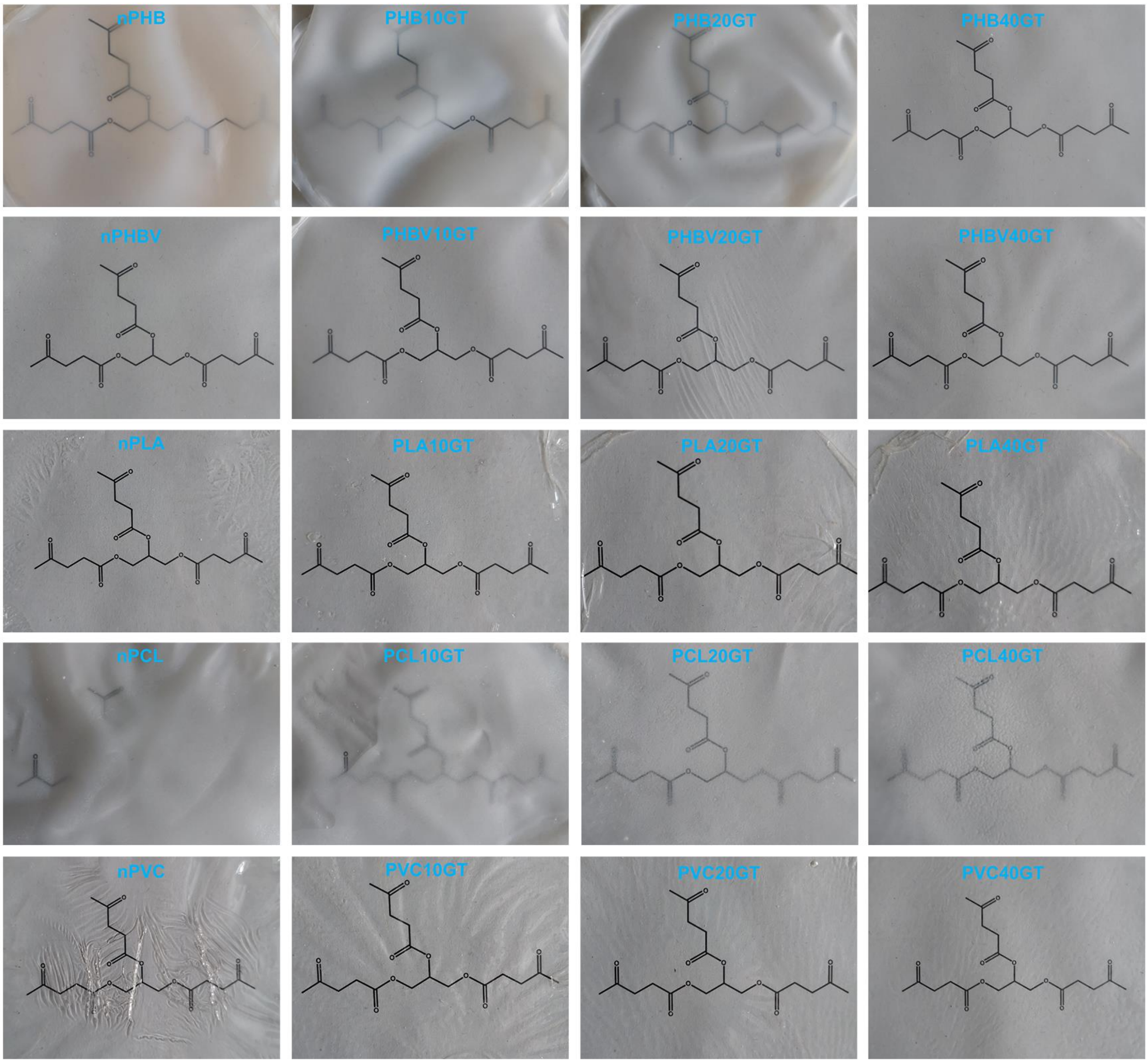

Figure S1. Photographs of the prepared neat polymeric films and with different plasticizer contents in order to have a qualitative evaluation of their transparency.

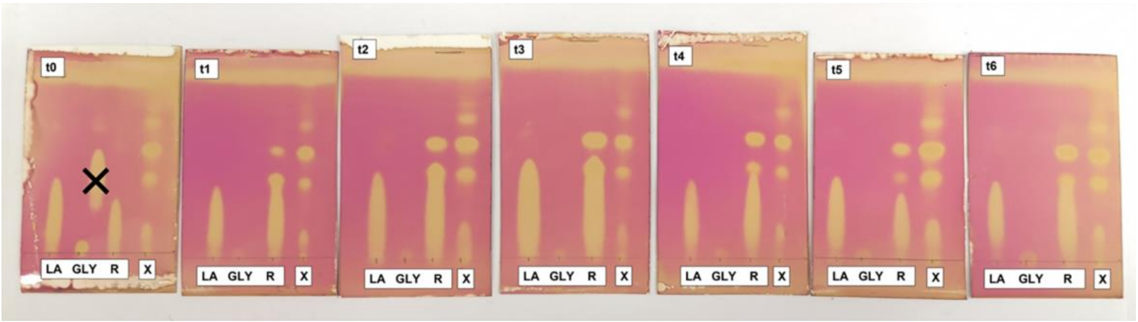

Figure S2. TLC plates of the reaction mixture from 0 hour to 6 hours. Reagents are named as *LA* and *GLY*, while the reaction mixture as *R*. *X* label indicates a product used as reference.

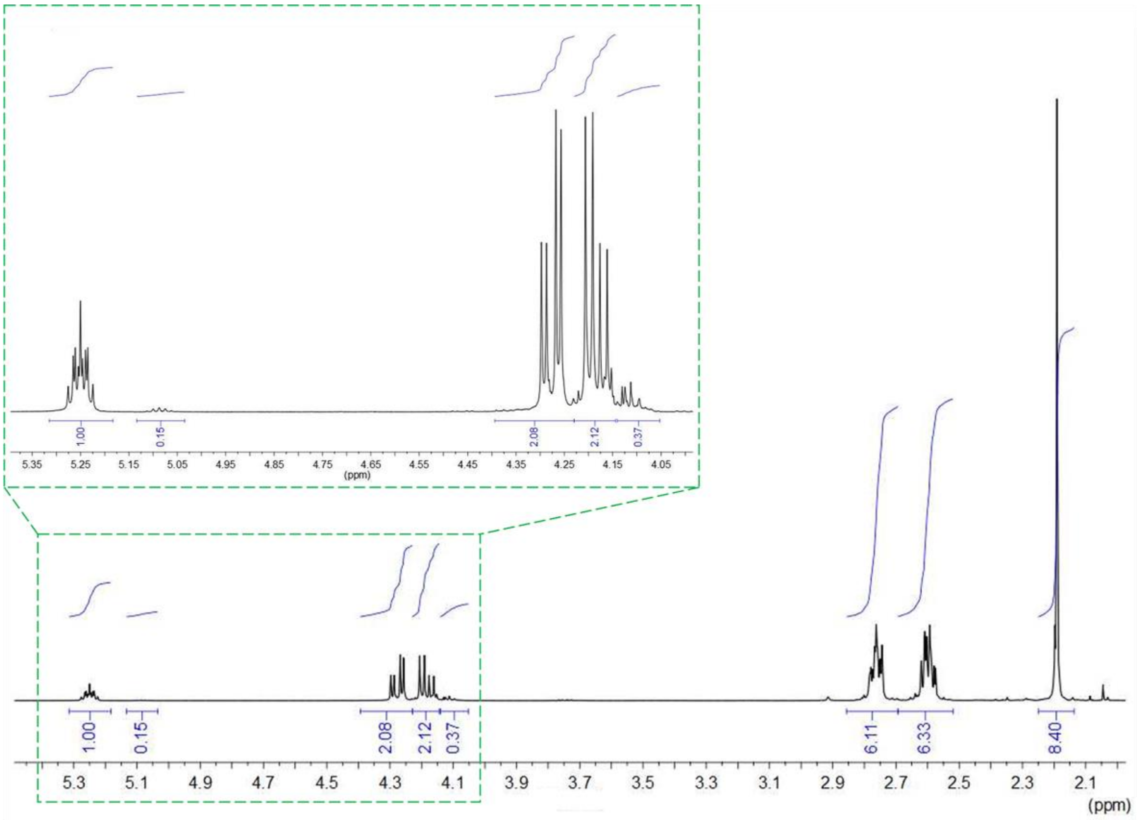

Figure S3. ^{1}H-NMR spectrum of GT plasticizer. The inset highlights the side peaks corresponding to unreacted hydroxyl groups.

^{1}H-and ^{13}C-NMR assignments of the final target product

[$CH_3C(=O)CH_2CH_2C(=O)O$]CH_2CH[$CH_3C(=O)CH_2CH_2C(=O)O$]CH_2[$CH_3C(=O)CH_2CH_2C(=O)O$]. The compound was obtained as a yellowish oil (70% yield).

^{1}H-NMR ($CDCl_3$, 400 MHz) A δ = 5.25 (m, 1H, CH*2*C*H*CH*2*), B δ=4.30−4.26 (m, 2H, $C(=O)OC\mathit{H_2}CHCH_2$), D δ = 2.82-2.71 (m, 2H, $CH_3C(=O)C\mathit{H_2}CH_2C(=O)O$), C δ = 2.65-2.57 (m, 2H, $CH_3C(=O)CH_2C\mathit{H_2}C(=O)O$), E δ = 2.29 (s, 3H, $C\mathit{H_3}C(=O)CH_2CH_2C(=O)O$).

^{13}C NMR ($CDCl_3$, 150 MHz) A δ=206.40 ($CH_3\mathit{C}(=O)CH_2CH_2C(=O)O$) B δ=172.23 ($CH_3C(=O)CH_2CH_2\mathit{C}(=O)O$) C δ=69.14 ($CH_2\mathit{C}HCH_2$) D δ=62,23 ($\mathit{C}H_2CH\mathit{C}H_2$) E δ=37.78 ($CH_3C(=O)CH_2\mathit{C}H_2C(=O)O$) F δ=29.73 ($\mathit{C}H_3C(=O)CH_2CH_2C(=O)O$) G δ=27.71 ($CH_3C(=O)\mathit{C}H_2CH_2C(=O)O$)

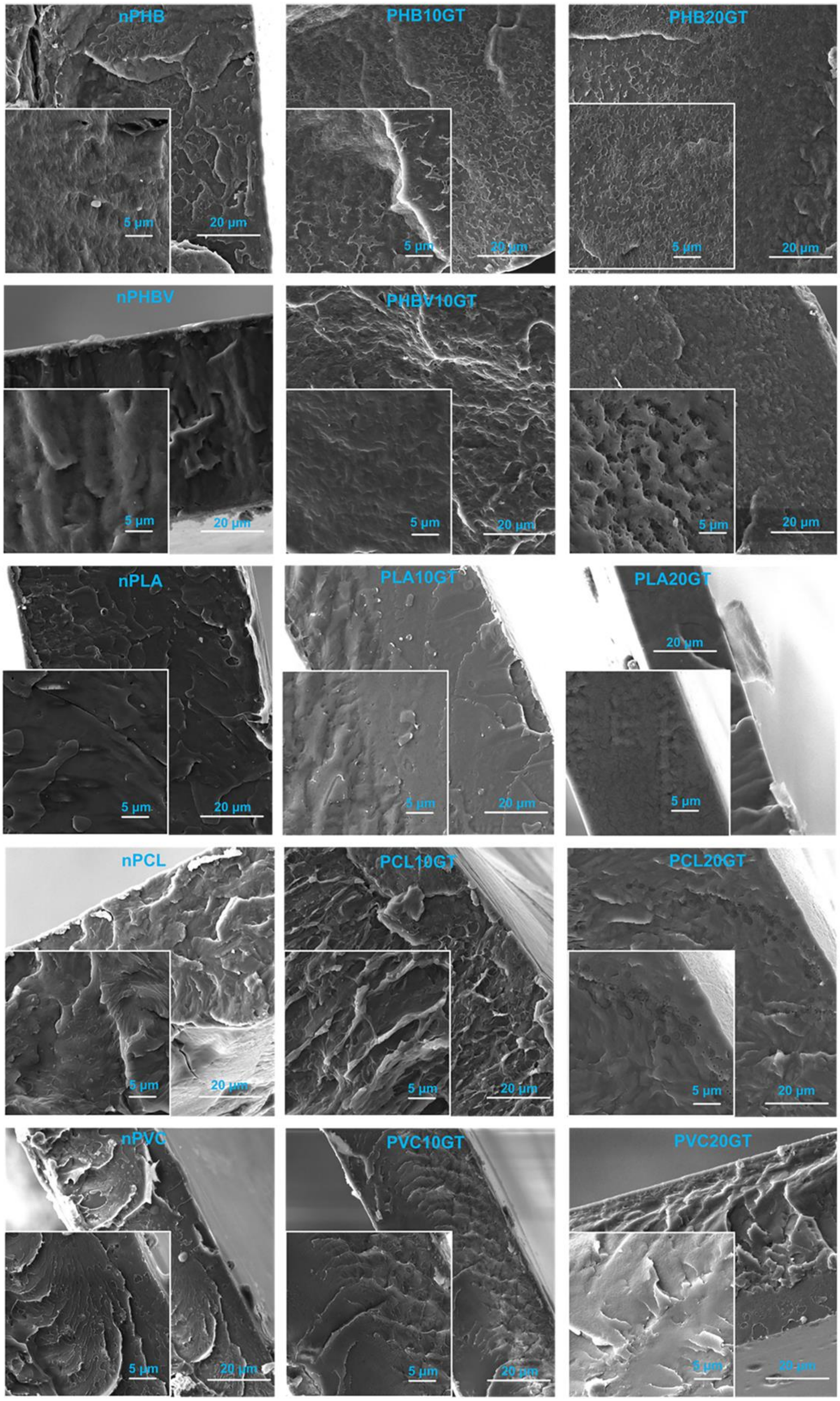

Figure S4. Cross section FE-SEM micrographs of neat and plasticized films. Higher magnifications are shown in the insets.

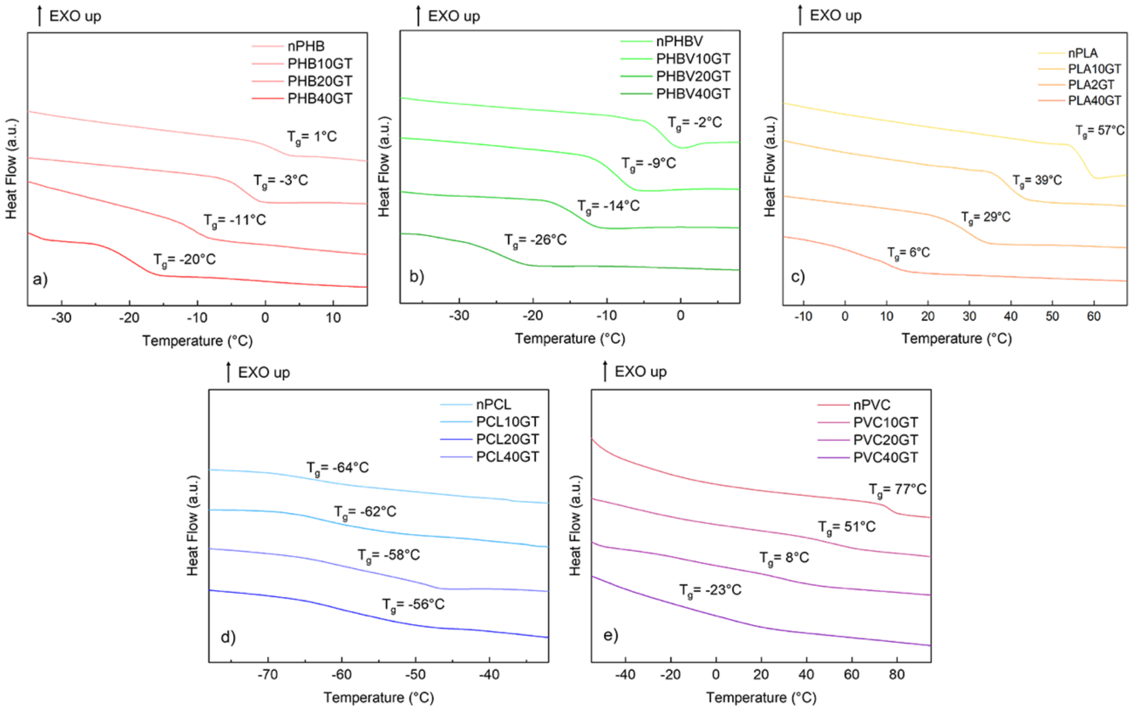

Figure S5. DSC thermograms recorded during the second heating scan at 20°C·min^{-1}, showing glass transition temperatures of neat and compounded formulations of (a) PHB (b), PHBV, (c) PLA, (d) PCL and (e) PVC.

Table S1. Glass transition temperature obtained from DSC analysis ($T_{g,DSC}$) and calculated by Fox equation ($T_{g,FOX}$), melting temperature (T_m) and melting enthalpy (ΔH_m) extrapolated from DSC thermograms, crystalline degree (X_c, calculated by Eq. 1), Young's modulus (*E*) and elongation at break (ε_{break}) obtained from tensile tests of neat and plasticized polymers. *E* and ε_{break} are expressed as mean value ± standard deviation.

**Polymer**	**GT content (phr)**	**$T_{g,DSC}$ (°C)**	**$T_{g,FOX}$ (°C)**	**T_m (°C)**	**ΔH_m (J/g)**	**X_c (%)**	***E* (MPa)**	**ε_{break} (%)**
PHB	0	1	1	175	79	54	1361±78	4±1
	10	-3	-5	169	77	52	1146±135	5±1
	20	-11	-11	164	71	48	944±67	15±4
	40	-20	-22	163	66	45	878±68	20±4
PHBV	0	-2	-2	-	-	-	586±83	15±2
	10	-9	-13	-	-	-	283±15	36±3
	20	-14	-8	-	-	-	260±59	21±7
	40	-26	-23	-	-	-	134±15	16±2
PLA	0	57	57	-	-	-	877±100	8±3
	10	39	42	-	-	-	321±19	372±63
	20	29	29	-	-	-	230±67	470±126
	40	6	4	-	-	-	25±15	311±12
PCL	0	-64	-64	62	83	60	274±5	8±1
	10	-62	-63	52	55	39	185±20	7±2
	20	-58	-61	50	50	36	140±26	8±2
	40	-56	-58	49	47	33	95±10	11±2
PVC	0	77	77	-	-	-	1214±189	3±1
	10	51	58	-	-	-	780±151	6±1
	20	8	41	-	-	-	589±193	74±3
	40	-23	12	-	-	-	132±14	446±133

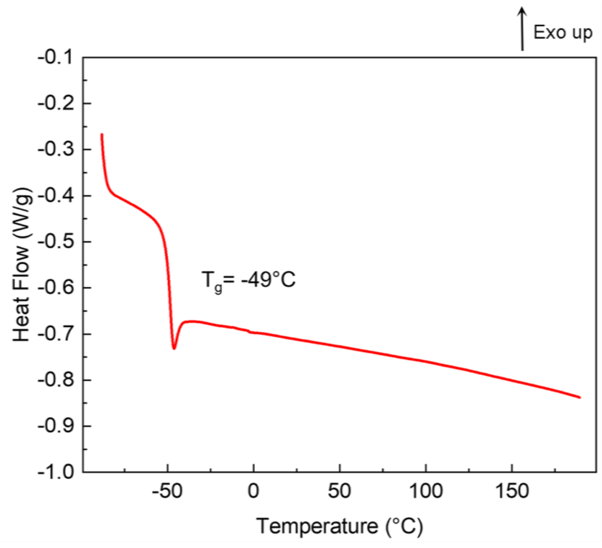

Figure S6. DSC thermogram of GT plasticizer recorded during the second heating scan using a scan rate of 20°C·min^{-1}.

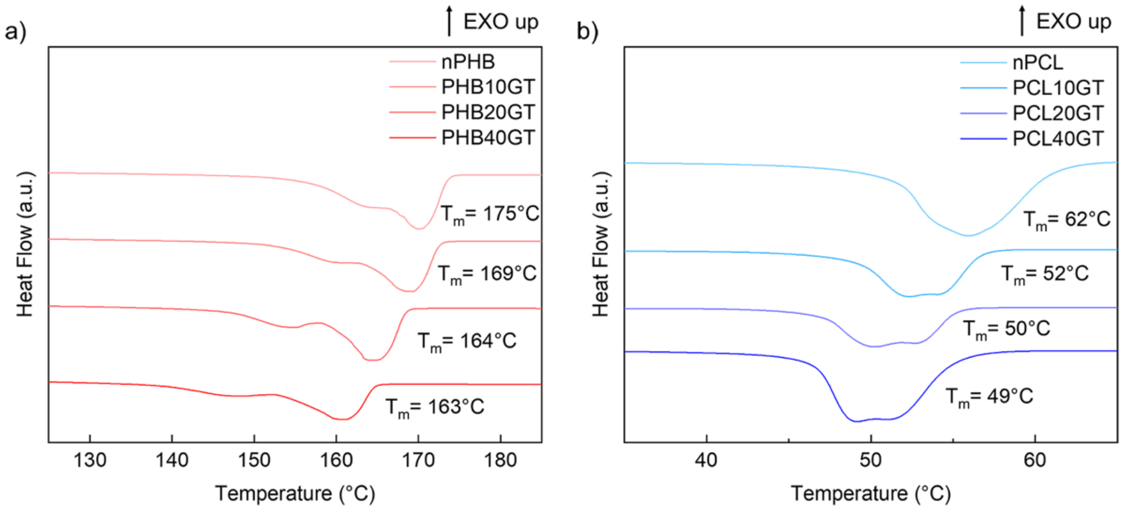

Figure S7. DSC thermograms recorded during the first heating scan at 10°C·min^{-1}, endothermic melting peaks and the relative melting temperatures of neat and compounded formulations of (a) PHB and (b) PCL.

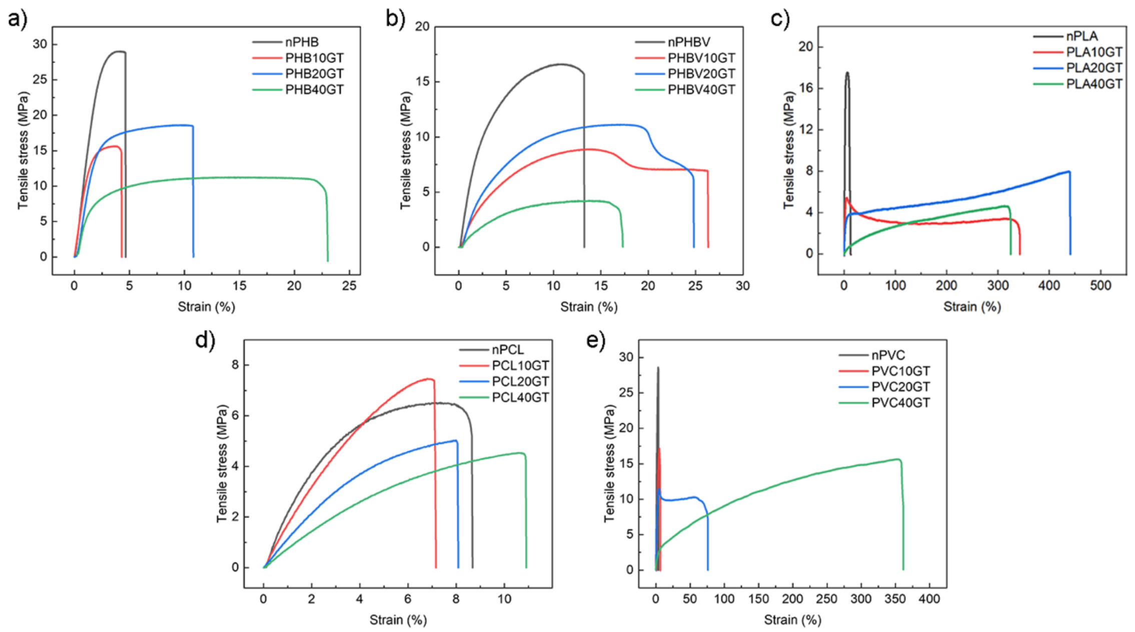

Figure S8. Stress-strain curves for neat and plasticized samples of (a) PHB, (b) PHBV, (c) PLA, (d) PCL and (e) PVC.

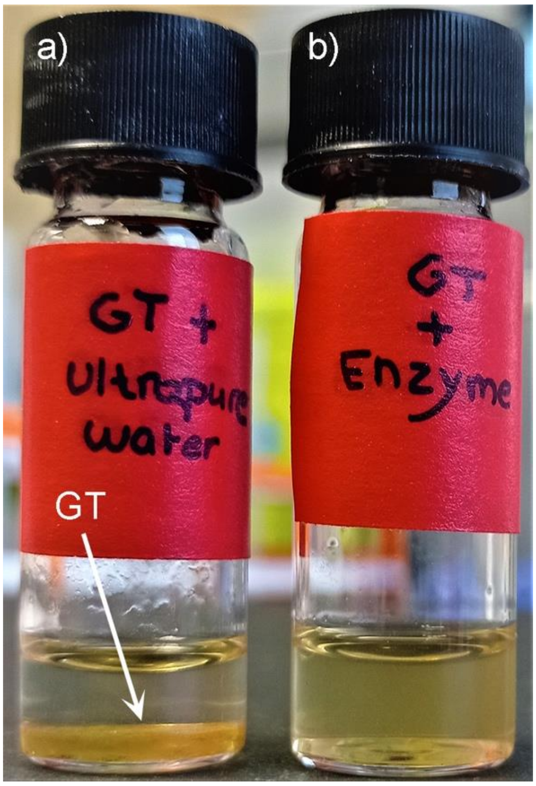

Figure S9. Incubation vials with plasticizer solubilized in (a) ultrapure water as control and (b) in ultrapure water with enzyme.
